# The value of melanoma inhibitory activity and LDH with melanoma patients in a Chinese population

**DOI:** 10.1097/MD.0000000000024840

**Published:** 2021-02-26

**Authors:** Chujun Li, Jinfang Liu, Lu Jiang, Jun Xu, Anjing Ren, Yu Lin, Gang Yao

**Affiliations:** a Department of Plastic and Burns Surgery; b Department of Oncology, The First Affiliated Hospital of Nanjing Medical University, Nanjing, China.

**Keywords:** lactate dehydrogenase, malignant melanoma, melanoma inhibitory activity, prognostic factor

## Abstract

Malignant melanoma is a highly malignant tumor originating from the melanocytes of the neural crest, which is prone to metastasis and has a poor prognosis. Previous research demonstrated that melanoma inhibitory activity (MIA) and lactate dehydrogenase (LDH) could serve as serum markers in malignant melanoma and indicate prognosis in the Caucasian race. Researchers suspected that both MIA and LDH could prompt the prognosis of malignant melanoma in the Chinese population. This study aimed to investigate the value of MIA and LDH in the prognosis of acral malignant melanoma.

From January 1, 2014, to December 31, 2017, in Jiangsu Province, 44 acral malignant melanoma patients with complete data were chosen from the clinic. The LDH levels were extracted from their clinical data, and MIA levels were measured by enzyme-linked immunosorbent assay method. 8 paired advancing samples before and after metastasis were examined. 22 health donors were matched to the patient group. Receiver operating characteristic (ROC) curves of MIA and LDH were drawn to determine acral malignant melanoma tumorigenesis and metastasis and finally got the cut-off value. Cumulative survival was illustrated with the Kaplan-Meier plot, and factors were compared using the Log-rank test.

Compared with age-matched healthy donors, MIA was significantly high in patients (*P* < .001). Moreover, serum MIA was significantly higher in III-IV stage patients than I-II stage patients (*P* < .001). However, there was no such association between LDH and melanoma stage and risk. Further study indicated that the MIA cut-off > 914.7pg/mL predicted disease progression with 86.4% specificity and 95.5% sensitivity. In the Kaplan-Meier analysis, MIA levels were independent risk factors for long-term mortality of acral malignant melanoma patients.

It concluded that the quantification of MIA in the serum should be performed as a general standard of care in patients at risk of developing metastatic melanoma.

## 1 Introduction

Malignant melanoma is a highly malignant tumor originating from the neural crest melanocytes, well-known for its high invasion and grim prognosis.^[1]^ In recent years, scientists have tried various new methods to judge the prognosis of tumors, including NGS Data,^[2]^ Raman-Enhanced Spectroscopy (RESpect) Probe,^[3]^ Ultrasound Analysis,^[4]^ and tumor markers. A tumor marker is a biomarker found in blood, urine, or body tissue that can be elevated by the presence of 1 or more types of cancer. The development of any cancer resulted from various factors, including changes at a molecular level.^[5,6]^ There are no precise biomarkers related to prognosis in melanoma; two biomarkers that may be related to the prognosis are melanoma inhibitory activity (MIA) and lactate dehydrogenase (LDH).

MIA was highly expressed in malignant melanocytes specifically. It is an 11 kDa protein that is strongly expressed and subsequently exocytosed by melanoma cells but not benign melanocytes.^[7]^ MIA interacts with MIA-interacting peptide ligands and fibronectin on the cell surface, which eventually induces migration of the melanoma cell.^[8]^ So far, studies in many countries have been conducted to verify the relationship between MIA and melanoma. However, research on the relationship between MIA levels and Chinese acral malignant melanoma patients is still blank.

LDH, an enzyme in the glycolytic pathway that converts pyruvate to lactate during hypoxia, is closely related to the occurrence, proliferation, and metastasis of several cancers.^[9]^ The latest American Joint Committee on Cancer melanoma TNM staging system recommends that metastatic melanoma patients be tested for LDH levels. The guidelines believe that LDH can be used as a serum marker to predict patients’ prognosis with metastatic melanoma.^[1]^

There have been long efforts to find effective(practical) markers to predict tumor recurrence and metastasis. MIA and LDH are proteins whose serum concentrations sensitively alter in response to the development and metastasis of melanoma, which have been widely studied and have been applied in clinical practice in the Caucasian race.^[10–14]^ However, in the Chinese race, the relevant data is still blank.

The purpose of the retrospective study was to evaluate the clinical value of 2 serological markers in melanoma: MIA and LDH. Serum MIA and LDH levels were measured in 22 I-II stage melanoma patients, 22 III-IV stage melanoma patients, and 22 health donors during follow-up for up to 3 years. Researchers gathered the information of disease stage and progression, presence and number of metastases, and overall survival (OS) for Statistical Analysis.

## 2 Materials and methods

### 2.1 Patient cohorts

All serum samples were obtained in acral malignant melanoma patients from the Department of Plastic and Burns Surgery and the Department of Oncology at the First Affiliated Hospital of Nanjing Medical University. The investigation was approved by the Helsinki Committee of the First Affiliated Hospital of Nanjing Medical University. All cases were confirmed by histopathology as melanoma and without other cancer, and no chemotherapy or radiotherapy history before inclusion in the study. 22 samples of age and sex-matched healthy donors were also collected in the survey. The definition of healthy donors were individuals who were free from cancer.

Researchers also evaluated changes in serum MIA and LDH in 8 patients with metastasis compared to their original levels (no metastasis period). The blood samples were gathered from patients at the first time of diagnosis. The patients were asked to review when the disease progress or at least once every 3 months. Blood samples were collected in tubes containing Ethylene Diamine Tetraacetic Acid and centrifuged for 5 minutes at 3000 rpm and the plasma samples were stored at -80°C until used.

### 2.2 Serum assays

Serum levels of MIA (R&D Systems, Quantikine, Human MIA) was evaluated with enzyme linked immunosorbent assays kits according to the manufacturers’ instructions. Clinical data of patients with LDH tested on their admission before and after tumor metastasis. These data were extracted and analyzed for tumor prognosis and survival situation.

### 2.3 Statistical analysis

Statistical analysis was conducted using SPSS 20.0 and Graphpad Prism 7.0 software. Numerical variables were presented as medians and interquartile ranges and categorical variables were shown as frequencies and percentages. Differences of measurement data and enumeration data were compared with Mann–Whitney *U* test and Kruskal-Wallis test respectively, and the correlation between groups was evaluated using the Spearman test. The sensitivity and specificity for MIA and LDH to predict disease progression were assessed by receiver operating characteristic (ROC) curves. To determine the risk of MIA or LDH for mortality in melanoma patients, the Kaplan-Meier plot was used to illustrate cumulative survival and the Log-rank test was performed to eliminate confounding factors. *P* < .05 was considered as statistically significant.

## 3 Results

**Figure 1:**
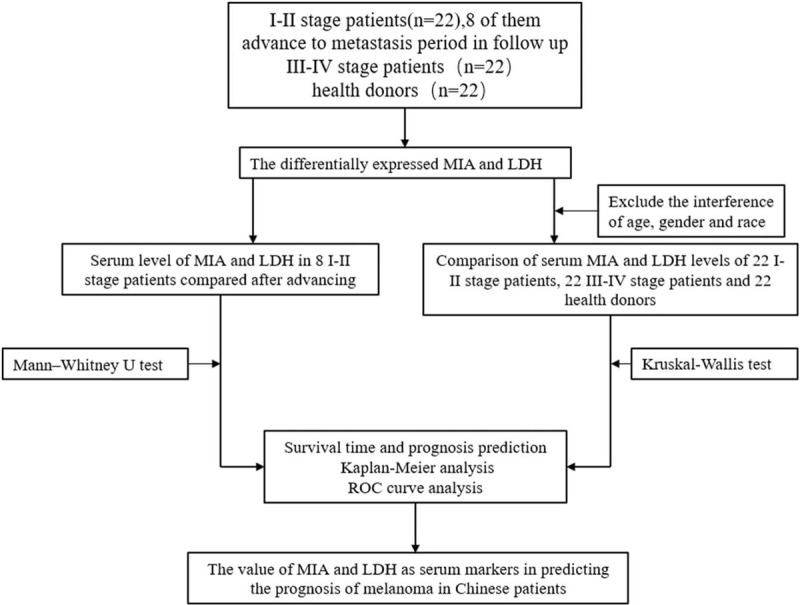
Overall design of the present research.

**Figure 2:**
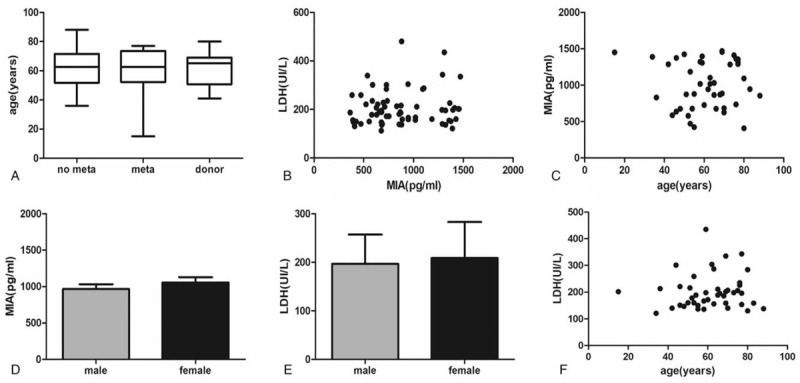
Comparison of age with no metastasis melanoma patients, metastasis melanoma patients, and health donors (A). Correlation between MIA and LDH(B). Comparison of MIA values with age(C) and gender (D). Comparison of LDH values with age (E) and gender (F).

The overall design and flowchart of this research is shown in Figure 1. Selected characteristics of the cases and controls are presented in Table 1. There was no difference among the I-II stage patients, III-IV stage patients, and the healthy donors in terms of gender and age. According to *t* test, neither MIA nor LDH were associated with age or gender (Fig. 2). All samples were run with no dilution. To verify the reproducibility of the assay, blood samples were all ran in duplicate. The mean level of MIA in III-IV stage melanoma patients was 1274.6 ± 175.1pg/ml, in I-II stage melanoma patients was 735.1 ± 184.9 pg/mL, and in healthy donors was 615.6 ± 169.6 pg/mL. Mean level of LDH from clinical data in III-IV stage melanoma patients was 212 ± 80.1 UI/L, in I-II stage melanoma patients was 192 ± 47.9 UI/L, and in health donors was 210 ± 83.8 UI/L (Table 2).

**Table 1:** Clinical characteristics of melanoma patients included in this study.

	Stage I/II disease (N = 22)	Stage III/IV disease (N = 22)	Healthy donors (N = 22)	*P* value
Age(mean ± SD)	62.1 ± 14.0	60.1 ± 15.7	61.0 ± 11.0	.97^1^
Gender (male)	14 (63.6%)	11(50.0%)	12 (54.5%)	.65^1^
Race (Chinese population)	22 (100%)	22 (100%)	22 (100%)	1.00^1^

^1^ Kruskal-Wallis test. SD = standard deviation.

**Table 2:** Serum marker levels in melanoma subgroups. (Mean±SD).

Serum marker	Stage I/II disease	Stage III/IV disease	Healthy donors	*P* value
MIA (pg/ml)	735.1 ± 184.9	1274.6 ± 175.1	615.6 ± 169.6	<0.0001^1^
LDH (UI/L)	192 ± 47.9	212 ± 80.1	210 ± 83.8	.89^1^
Number	22	22	22	

^1^Kruskal-Wallis test. LDH = lactate dehydrogenase, MIA = melanoma inhibitory activity, SD = standard deviation.

**Figure 3:**
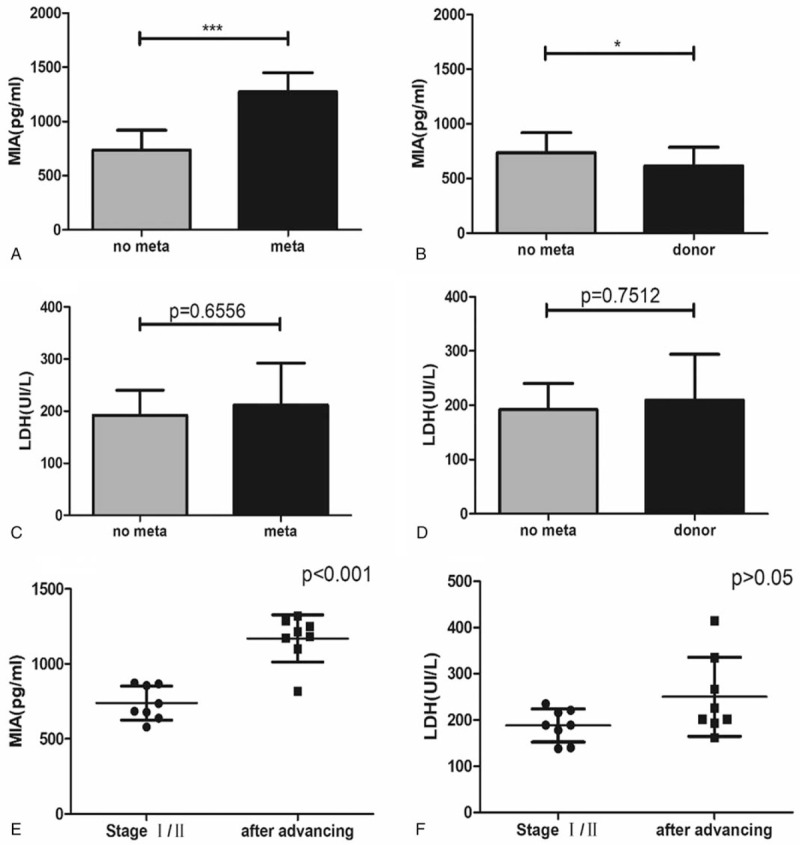
Comparison of MIA values with no metastasis patients and metastasis patients (A), no metastasis patients, and health donors (B). Comparison of LDH values with no metastasis patients and metastasis patients (C), no metastasis patients and health donors (D). Comparison of MIA changes in 8 patients with melanoma before and after metastasis (E). Comparison of LDH changes in 8 patients with melanoma before and after metastasis (F).

Significantly higher concentrations of MIA were found in III-IV stage patients than in I-II stage melanoma patients (Fig. 3A). However, there was no significant difference between the level of LDH in III-IV stage and I-II stage melanoma patients (Fig. 3C). Similarly, the MIA levels of I-II stage patients were also significantly higher than those of the healthy donors, while the level of LDH had no significant difference (Figure 3B, D).

The changes in MIA and LDH levels for eight patients who ultimately developed hepatic or lung metastases were illustrated in Table 3. Compared to the serum level of MIA before metastasis, the level of MIA elevated significantly, but there was no statistical difference in LDH (Fig. 3E, F).

**Table 3:** Serum marker levels in Stage I/II no metastasis patients after advancing.

Serum marker	Stage I/II disease	Stage I/II disease after advancing	*P* value
MIA (pg/ml)	738.9 ± 113.5	1169.2 ± 157.6	.0011^1^
LDH (UI/L)	188 ± 35.8	250 ± 85.3	.10^1^
Number	8	8	

^1^Mann–Whitney *U* test. LDH = lactate dehydrogenase, MIA = melanoma inhibitory activity.

**Figure 4:**
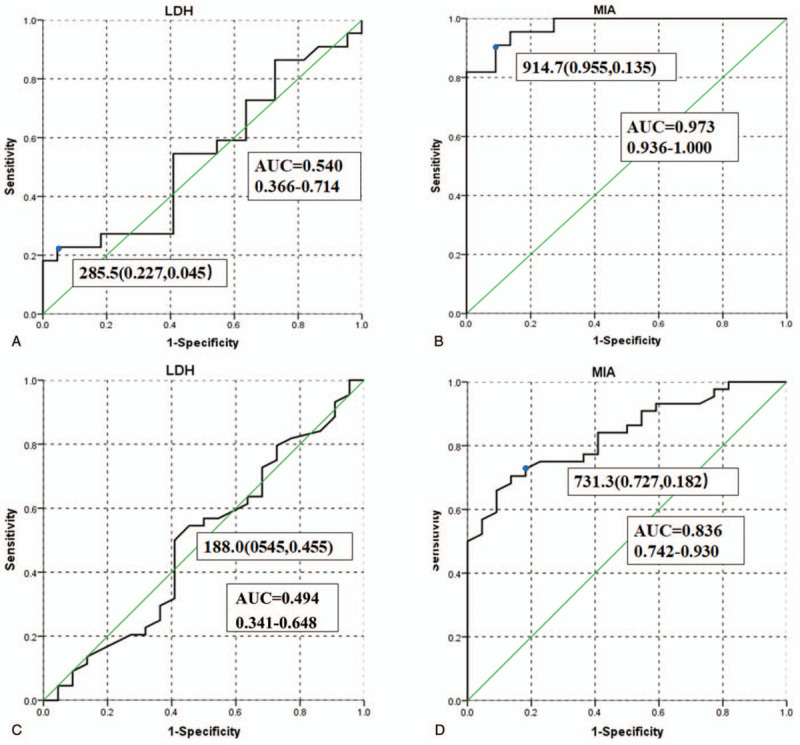
ROC curve analysis: LDH cutoff point between melanoma patients in Stage I/II disease and patients in stage III/IV disease (A), MIA cutoff point between melanoma patients in Stage I/II disease and patients in stage III/IV disease (B), LDH cutoff point between patients with melanoma or not (C), MIA cutoff point between patients with melanoma or not (D).

ROC curves were applied to compare the marker levels of III-IV stage patients to I-II stage patients. The area under concentration curve (AUC) of MIA was 0.973 with 95.5% sensitivity and 86.4% specificity (cut-off value: 914.7 pg/mL); The AUC of LDH was 0.54 with 22.7% sensitivity and 95.5% specificity (cut-off value: 285.5UI/L). Additionally, ROC analysis was also conducted for marker levels of melanoma patients compared to healthy donors. The AUC of MIA was 0.836 with 72.7% sensitivity and 81.8% specificity (cut-off value: 731.3pg /ml); The AUC of LDH was 0.494 with 54.5% sensitivity and 54.5% specificity (cut-off value: 188.0UI/L). The comparisons were shown in Figure 4.

**Figure 5:**
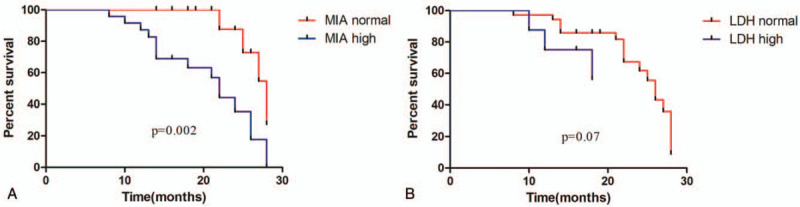
Factors associated with mortality (Kaplan-Meier survival estimates): Kaplan-Meier survival estimates according to whether MIA greater than or equal to 914.7pg/ml; According to the cutoff value, 25 patients were classified as MIA high group and 19 patients were classified as MIA normal group (A). Kaplan-Meier survival estimates according to whether LDH greater than or equal to 285.5UI/L; According to the cutoff value, 8 patients were classified as LDH high group, and 36 patients were classified as LDH normal group (B).

To determine whether MIA or LDH values have associations with the prognosis of patients, the Kaplan-Meier survival estimates were applied. MIA affected prognosis. The median survival of patients with MIA ≥ 914.7 pg/mL was 22 months, which was 28 months of patients with MIA < 914.7 pg/mL (*P* = .002, HR = 0.2223, 95% CI: 0.08577–0.5762, Fig. 5A). However, LDH did not affect prognosis, there was no statistically significance of median survival data between patients with LDH≥ 285.5UI/L or below (*P* = .07, HR = 0.1625, 95% CI: 0.02274–1.161, Fig. 5B).

## 4 Discussion

The clinical classification of malignant melanoma is divided into 4 types: superficial spreading melanoma, nodular melanoma, acral lentiginous melanoma, and lentigo maligna melanoma. Melanoma, whose incidence has been increasing steadily over the past 30 years, is a kind of tumor with inadequate response to chemotherapy and radiation therapy.^[15,16]^ Malignant melanoma has a grim prognosis after the onset of metastasis. Given the higher survival rate of melanoma when early detection of the disease occurs, accurate diagnostic tests for early detection of melanoma will be beneficial. Besides, as mortality increases dramatically and the disease develops rapidly, it is necessary to find reliable tumor markers to detect melanoma metastasis and monitor responses to therapy.^[17]^

In previous clinical research, Researchers found that the proportion of pathological type in melanoma results from race difference.^[1,18,19]^ Moreover, the proportion of pathological types of melanoma among different races varies.^[20,21]^ The researchers designed this experiment to verify whether serum markers such as MIA and LDH can play a role in prompting metastasis and prognosis in Chinese races.

It has been reported that serum LDH was valuable in diagnosing tumors and predicting the progression of many cancer types, such as lung cancer, breast cancer, and prostate cancer.^[22–24]^ In recent years, many studies focused on the diagnosis and prognostic value of LDH on melanoma. Wagner et al observed that LDH might be an independent prognostic factor for response to anti-PD-1 or combined anti-PD-1 plus anti-CTLA-4 antibodies therapy and OS of melanoma patients.^[25]^ Moreover, Kelderman et al found that for melanoma patients whose serum LDH level is more significant than twice the upper limit of normal, long-term treatment with ipilimumab may not benefit.^[26]^ To conclude, LDH has certain predictive value for the treatment and prognosis of patients with malignant melanoma. Our study also performed Kaplan-Meier survival analysis based on patients’ LDH levels, but did not find a relationship between LDH levels and prognosis. This result may be due to the small sample size of patients included in this study. Only 8 patients were included in the LDH high group, which caused statistical errors.

In 1989, MIA was first isolated from malignant melanoma cells and has been extensively studied for the value of treatment and prognosis of malignant melanoma.^[27]^ A study conducted by Hofmann et al demonstrated that patients with higher serum MIA concentration had worse disease-free survival and OS.^[28]^ Odashiro et al measured serum MIA levels in patients with metastatic malignant melanoma, non-metastatic malignant melanoma, and healthy controls, respectively. No significant difference in serum levels of MIA between healthy controls and non-metastatic malignant melanoma was obtained in our study. Still, the difference in MIA expression was statistically significant in the healthy control group and metastatic malignant melanoma group, non-metastatic and metastatic malignant melanoma group.^[29]^ Tas et al observed that there was a trend for worsened prognosis in patients with rising MIA level.^[30]^

All the studies above have shown that MIA may be a prognostic marker for malignant melanoma. Therefore, our hypothesis, which we have confirmed through this study, was that MIA serum levels were statistically significantly elevated in patients with metastatic melanoma. As a potential biomarker for metastasis, the specificity was 86.4%, and sensitivity was 95.5%. This indicates that MIA as a serum marker also plays a role in predicting the pathogenesis and metastasis of malignant melanoma in the Chinese population.

There are still several shortcomings in this article. For a serum marker for multiple tumors, LDH is thought to have the function of predicting tumor prognosis,^[31]^ but in this study there was no significant difference in serum levels in the I-II stage group, III-IV stage group, and healthy control group. The effects of LDH levels on prognosis in melanoma patients were not statistically different. This may be due to the low sample size included in this study. Especially in the study, samples of LDH high group were only 8, which led to a statistical error in the results. Several problems were also found in the research that experimental results showed the level of MIA in Chinese melanoma patients is much lower than that in Caucasians in the same experiment. Researchers duplicated the experiment, tried to dilute the sample, and got the same result. This diversity may due to the differences in MIA concerned gene involved in the development of melanoma among different races. This conjecture requires further molecular mechanism experiments and larger sample clinical data collection to verify. According to clarify the molecular mechanisms of differential genes, the pathogenesis between different pathological types in melanoma can be understood.

## Author contributions

**Data curation:** Jun Xu, Anjing Ren, Yu Lin.

**Funding acquisition:** Chujun Li.

**Investigation:** Chujun Li, Jinfang Liu.

**Methodology:** Chujun Li, Lu Jiang.

**Project administration:** Chujun Li.

**Resources:** Chujun Li, Gang Yao.

**Software:** Chujun Li.

**Supervision:** Gang Yao.

**Writing – original draft:** Chujun Li, Lu Jiang.

**Writing – review & editing:** Chujun Li, Jinfang Liu.
